# Transcriptome analysis of *megalurothrips usitatus* (Bagnall) identifies olfactory genes with ligands binding characteristics of MusiOBP1 and MusiCSP1

**DOI:** 10.3389/fphys.2022.978534

**Published:** 2022-09-26

**Authors:** Zhaoyang Li, Weiyi Chen, Xiaoshuang Wang, Wen Sang, Huipeng Pan, Shaukat Ali, Liangde Tang, Jianhui Wu

**Affiliations:** ^1^ Key Laboratory of Bio-Pesticide Innovation and Application, Engineering Research Center of Biological Control, College of Plant Protection, South China Agricultural University, Guangzhou, China; ^2^ State Key Laboratory Breeding Base of Green Pesticide and Agricultural Bioengineering, Key Laboratory of Green Pesticide and Agricultural Bioengineering, Ministry of Education, Guizhou University, Guiyang, China

**Keywords:** *Megalurothrips usitatus* (Bagnall), olfactory proteins, binding assay, illumina sequencing, expression pattern

## Abstract

The olfactory system is an important component of insect behavior and is vital for survival and reproduction. However, the genomic characterization and molecular basis of the olfactory response of *Megalurothrips usitatus* remain relatively unknown. RNA sequencing-built developmental transcriptomes of nymphs, pupae, and adult *M. usitatus* were examined in order to establish the sequence-based background of *M. usitatus* olfactory responses. A total of 56,669 unigenes were annotated using GO, NR, Pfam, eggNOG, SwissProt, and KEGG. The number of differentially expressed genes between pupae and nymphs, males and nymphs, and females and nymphs were 10,498, 9,235, and 10,964, respectively. One odorant-binding protein (MusiOBP1) and one chemosensory protein (MusiCSP1) were selected from the transcriptome, and their full-length sequences were obtained using RACE PCR. The relative expression of MusiOBP1 was the highest in primordial females, whereas the relative expression of MusiCSP1 was the highest in primordial pupae. The strongest binding ability to the odor-binding protein MusiOBP1 was observed for β-citronellol. 3-Hydroxy-2-methyl-4-pyrone showed the strongest binding affinity to MusiCSP1. Our analysis suggests that MusiOBP1 and MusiCSP1 may play significant roles in mediating *M. usitatus* host recognition. This research will improve our knowledge of odorant-binding proteins and chemosensory proteins, which will in turn improve our understanding of insect olfactory systems.

## Introduction

The insect olfactory system plays an important role in behavior related to survival and reproduction, including foraging, couple searching, and searching for ovipositional sites (Field et al., 2008; Carey et al., 2010; Li F. Q. et al., 2019a). Odorant-binding proteins (OBPs) and chemosensory proteins (CSPs) transport odorant molecules into the lymphin sensilla and reach odorant receptors located on the dendrites of olfactory receptor neurons, resulting in relevant olfactory behavioral responses (Zhang et al., 2020a; Ullah et al., 2020; Cui et al., 2022).

OBPs are water-soluble proteins composed of 130–140 amino acids, approximately 14–17 KD with an isoelectric point of 4–5. There are six conserved cysteines in a typical OBP signal peptide sequence; some insects are type ‘Plus-C’ with more than six cysteines, type ‘Minus-C’ with less than six cysteines, and ‘Atypical’ OBPs with an extended C-terminal region (Zhou et al., 2008). CSPs are small soluble proteins, consisting of 100–115 amino acids, typically characterized by four conserved cysteine residues that form two disulfide bonds and then compose two small rings to maintain a stable configuration (Picimbon et al., 2000). More attention has been paid to the use of OBPs than CSPs as targets to discover behaviorally active compounds as well as novel bioinspired synthetic attractants and repellents for pest management (Thireou et al., 2018; Venthur et al., 2018; Xie et al., 2019). Some CSPs exhibit binding activity to plant volatiles and sex pheromones (Gu et al., 2012; Zeng et al., 2018).

The rapid development of transcriptomics provides a new direction for mining insect olfactory genes (Jia et al., 2016; Scieuzo et al., 2021). For example, the antennal transcriptome of Manduca sexta identified unique putative OBP-coding transcripts and 14 additional CSP-coding gene fragments (Grosse-Wilde et al., 2011), providing a new methodology for the functional study of olfactory proteins. Thrips (order Thysanoptera), which include approximately 7,700 species, constitutes a huge insect group, approximately 1% of which are important agricultural pests (Mound 2009a); however, we still lack a fundamental understanding of their olfactory system.

*Megalurothrips usitatus* is a major insect pest that is prevalent in subtropical regions worldwide, including southern China, New Zealand, and Malaysia (Mound and Walker 1987; Mound and Azidah 2009b; Dialoke 2013; Yan et al., 2017). *M. usitatus* can feed on 49 host plants, of which 32 belong to Leguminosae and others include Poaceae Barnhart, Solanaceae Juss., Rutaceae, and Brassicaceae (Li et al., 2019c). It can damage host plants throughout its growth period and prefers to feed on flowers and fruits, seriously affecting both the yield quality and commodity value (Fan et al., 2013). In recent years, studies on *M. usitatus* have mainly focused on its growth and development (Qiu et al., 2014), occurrence dynamics (Tang L. D. et al., 2015b), and physical and chemical controls (Luo et al., 2014; Shen et al., 2017). The damage caused by *M. usitatus* is increasing annually in southern China, and its control mainly relies on chemical insecticides (Sang et al., 2014; Xiao et al., 2014). The intensive use of chemical insecticides, such as emamectin benzoate, spinetoram, and beta-cypermethrin, against *M. usitatus* has led to the development of resistance (Tang et al., 2016; Khan et al., 2021), which increases the required dose and application frequency, resulting in environmental and food safety hazards.

However, olfactory-related proteins have not been studied in *M. usitatus*. Clarifying the molecular mechanisms of the olfactory system and compound selection are vital for promoting future integrated pest control strategies. In this study, *M. usitatus* transcriptomes were obtained using RNA-seq. We identified differentially expressed genes (DEGs) among different developmental stages, as well as one OBP gene (MusiOBP1) and one CSP gene (MusiCSP1) were identified and obtained in full-length. Fluorescence binding assay analysis was carried out on the binding characteristics of ten odorous ligands. Our results enrich the theoretical basis for clarifying the chemical communication between *M. usitatus* and its environment, which can be used to explore new behavioral control methods.

## Materials and methods

### Insect cultures

*M. usitatus* was originally collected from approximately 3,000 individuals from the cowpea planting area of Nanbin Farm, Sanya City, Hainan Province, China in March 2019. In the laboratory, *M. usitatus* was continuously cultured in an artificial incubator at 26 ± 1°C, 65 ± 5% relative humidity, and a 14:10 h light: dark cycle for more than 2 years. Samples of *M. usitatus* were taken from the laboratory population.

### Construction of cDNA library and illumina sequencing

Each developmental stage of *M. usitatus* was selected for research, and a total of four groups of samples were collected: the first and second instar nymphs at a ratio of 500:500 were combined into the nymph group, 500 pre-pupa and 500 pseudo-pupa were combined into the pupa group, and 1,000 female and 1,000 male adults were divided into two groups respectively. Three replicates were performed for each group. Total RNA was extracted using an RNA microextraction kit (Magen, China) and treated with DNase I. mRNA with polyA structure was enriched using oligo (DT) magnetic beads, and then fragmentation buffer was added to break the mRNA into short pieces. The first strand of cDNA was synthesized with 6-base random primers and reverse transcriptase using short mRNA fragments, and dNTPs and DNA polymerase I were added to synthesize the second strand of cDNA. The cDNA library was constructed, followed by library fragment enrichment by PCR amplification, and the constructed library was detected using the Agilent 2,100 (Agilent Technologies, United States ) and ABI StepOnePlus Real-Time PCR System. The library was sequenced using the Illumina Hiseq2000 platform. Transcriptome data were reanalyzed by Suzhou PANOMIX Biomedical Tech Co., Ltd.

### Transcriptome data analysis and identification of DEGs

After the samples were sequenced using the Illumina sequencing platform and raw data were obtained, the filter was combined with low-quality reads to obtain clean reads. The original offline data were filtered and reads with joints, lengths < 50 bp, and average sequence quality < Q20 were removed. Unigenes were obtained using Trinity software for *de novo* assembly of clean reads (Grabherr et al., 2011). These unigenes were then used for BLAST searches with annotations against GO, NR, KEGG, eggNOG, SwissProt, and Pfam. We used a false discovery rate ≤ 0.001 and an absolute value of log2 fold > 2 as thresholds to determine the difference in gene expression. DEGseq and annotation were used to identify gene expression at two different developmental stages (Table 1). According to the initial FPKM value of each development sample, we used log2 with FPKM + 1 for standardized calculation and created a heat map and a trend analysis map.

**TABLE 1:** OBP and CSP genes at four developmental stages.

Gene ID		Annotation
OBPs	TRINITY_DN486_c9_g1	Swissport: OBP72_ANOGA General odorant-binding protein 72 (Fragment) OS = *Anopheles gambiae* OX = 7165 GN = Obp72 PE = 3 SV = 1
	TRINITY_DN10890_c1_g1	NR: XP_026290294.1 general odorant-binding protein 83a-like (Frankliniella occidentalis) &gt;AEP27187.1 pheromone-binding protein (Frankliniella occidentalis)
	TRINITY_DN671_c0_g1	NR: XP_034238437.1 general odorant-binding protein 72-like (Thrips palmi)
	TRINITY_DN25247_c0_g1	NR: XP_026283043.1 general odorant-binding protein 70 (Frankliniella occidentalis)
	TRINITY_DN3336_c1_g1	NR: XP_034232292.1 general odorant-binding protein 56 days-like isoform X1 (Thrips palmi)
	TRINITY_DN12344_c0_g1	NR: XP_026276657.1 general odorant-binding protein 19 days-like isoform X1 (Frankliniella occidentalis)
CSPs	TRINITY_DN1806_c10_g1	NR: AEP27186.1 chemosensory protein (Frankliniella occidentalis)
	TRINITY_DN12423_c0_g1	NR: QSB36976.1 chemosensory protein 2 (Frankliniella intonsa)

### Amplification of known sequences of MusiOBP1 and MusiCSP1

To preliminarily study the function of olfactory proteins, TRINI-TY_DN10890_c1_g1 and TRINITY_DN1806_c10_g1 were selected in one clustal, which could be NR annotated to Frankliniella occidentalis, and known sequences were amplified. The primers used are listed in Supplementary Table S1. PCR amplification, product purification, and recovery were performed using agarose gel electrophoresis. The recovered product was ligated and transformed using the pEASY® -T1 simple cloning vector. Single colonies were selected and cultured overnight in 5 ml Luria-Bertani (LB) liquid medium (containing 100 mg/L ampicillin) in a shaker set at 200 rpm and 37°C. Positive clones were selected for the bacterial solution via colony PCR (Jin et al., 2008) and sent to San-gon Biotech Co., Ltd. (Shanghai, China) for sequencing.

### Acquisition and analysis of full-length sequences of MusiOBP1 and MusiCSP1

Gene-specific primers were designed using Primer Premier 6.0, according to the olfactory-related protein gene fragment sequences; the primer sequences are listed in Supplementary Table S2. To synthesize the 3’ and 5’ RACE Ready cDNA, PCR was performed for the 5’ and 3’-full rapid amplification of the cDNA end-core set using the Smart RACE cDNA amplification kit (Clontech, 634923). Detection was performed using 1% agarose gel electrophoresis after PCR, and the TIANgel Midi Purification Kit was used to recover the DNA fragments. The PCR recovery products linked to the pEASY-T1 vector and transformation, positive clone identification, and sequencing were the same as those described in Section 2.3. The intermediate fragment, 3’ sequence, and 5' sequence of each target gene were spliced using Geneious 7.1.4, and repeated regions in the sequence were removed to obtain full-length MusiOBP1 and MusiCSP1 cDNA sequences. A phylogenetic tree was constructed using the neighbor-joining (NJ) software Mega 7 and was repeated 1,000 times. The online biology website ExPASY (http://web.expasy.org/compute_pi/) was used to predict protein molecular weight and iso-electric point. SignaIP 5.0 (https://services.healthtech.dtu.dk/service.php?SignalP-5.0) was utilized for signal peptide prediction.

### Study of the expression pattern of MusiOBP1 and MusiCSP1

To clarify MusiOBP1 and MusiCSP1 expression at each developmental stage (first instar nymphs, second instar nymphs, third instar nymphs (pre-pupae), forth instar nymphs (pseudo-pupae), and primary eclosion females and males), 200 insects were collected at each stage and used for RNA extraction. Three replicates were carried out for each experiment. First-strand cDNA was synthesized using a PrimeScript™ RT Reagent Kit with gDNA Eraser (Perfect Real Time). RT-qPCR (Bio-Rad CFX96, United States ) was performed using the PerfectStartTM Green qPCR SuperMix and the following conditions: 95°C for 30 s, 40 cycles at 94°C for 5 s, 55°C for 30 s, and 72°C for 10 s. GAPDH from *M. usitatus* was used as the reference gene and the primers used are listed in Supplementary Table S3. Laboratory studies have shown that GAPDH is an internal reference gene suitable for different developmental stages. The relative expression level was defined as a unit, using the expression level of freshly emerging males as a control. The CFX manager software was used to analyze the required expression levels of genes at different ages, and one-way analysis of variance (ANOVA) was used to analyze the significance using SPSS (v. 21.0; IBM Corp., Armonk, NY, United States ) and GraphPad Prism (v. 8.0.2) to create the graphs.

### Gene cloning of MusiOBP1 and MusiCSP1

The open reading frame of the olfactory-related protein gene was amplified and the primer sequences are listed in Supplementary Table S4. The amplified PCR product was transferred to a pEASY-T1 cloning vector. After PCR detection and sequencing, monoclonal colonies with high expression levels were cultured in LB liquid medium. Plasmids were extracted using a plasmid extraction kit [Tiangen Biotech (Beijing) Co., Ltd., Beijing, China]. QuickCut™ BamHI [Takara Biomedical Technology (Beijing) Co., Ltd., China] and QuickCut™ HindIII [Takara Biomedical Technology (Beijing) Co., Ltd., China] were used for double enzyme digestion of the recombinant cloned plasmid and the expression vector pET32a(+). After electrophoretic recovery, T4 ligase was used to ligate and transform the recovered products. The operational steps of the colony PCR were sent to Sangon Biotech (Shanghai) Co., Ltd., China for sequencing to select positive clones and plasmid extraction using the TIANprep Mini Plasmid Kit [Tiangen Biotech (Beijing) Co., Ltd.].

### Protein expression and purification

The transformation steps of the recombinant expression plasmid and empty plasmid into *Escherichia coli* BL21(DE3) competent cells were the same as described above. Single colonies were selected and cultured overnight in 5 ml LB liquid medium (containing 100 mg/L ampicillin) in a shaker set at 200 rpm and 37°C. This was then transferred into LB liquid medium at a ratio of 1:100, cultured at 37°C in an oscillator at 200 r/min, and when the OD 600 value reached 0.5, isopropyl-β-D-thiogalactopyranoside was added to make a final concentration of 1 mmol/l. The culture was then induced in a shaker at 30°C for 10 h. After centrifugation at 4°C for 30 min at 10,000 rpm, the bacterial precipitates were collected and resuspended in ultrasonic lysate buffer (50 mM Tris-HCl, 500 mM NaCl, pH 8.0). The expression products were analyzed using 10% SDS-polyacrylamide gel electrophoresis (SDS-PAGE) to select bacterial cells with the correct band size for many expressions. The ultrasonic crusher circulated and broke bacterial cells for approximately 15 min by repeatedly running for 5 s and stopping for 5 s. Ni-NTA Sepharose Purification Res-in (Sangon Biotech (Shanghai) Co., Ltd., China) was added to the Ploy-Prep® chromatography columns (Bio-Rad), and the target protein was purified using the gravity method. The histidine-tagged protein was eluted on the column with elution buffer (20 mM Tris-HCl, 500 mM NaCl, pH 7.4) containing different imidazole concentrations (50, 100, 200, and 300 mmol/l) at twice the column volume.

The primary antibodies, ProteinFind^®^ Anti-His Mouse Monoclonal Antibody (TransGen Biotech Co., Ltd., Code#HT501), and secondary antibody, ProteinFind^®^ Goat Anti-Mouse IgG (H + L), HRP Conjugate (TransGen Biotech Co., Ltd., Code#HS201) were used for western blotting analysis. The NBT/BCIP color-developing solution was configured at a ratio of 1:1, and the solution was left in the dark at room temperature. The color was allowed to develop at room temperature on a shaker for 5 min, and images were taken on a chemical imager.

### Enzyme digestion and purification of recombinant protein

The purified target protein eluate was transferred to a dialysis bag, placed in rEK buffer (250 mM Tris-HCl, 50 mM NaCl, 2.5 mM CaCl2, pH 7.4), and slowly dialyzed in a refrigerator at 4 °C for 12 h. Recombinant bovine enterokinase light chain, yeast-derived [Sangon Biotech (Shanghai) Co., Ltd., code #C520305] was used to cut off the His-tag of the fusion protein. The resulting protein solution was re-purified. The purified protein was transferred to a dialysis bag, placed in PBS (pH 7.4), and slowly dialyzed in a refrigerator at 4°C for 12 h. A vacuum concentrator was used to concentrate the target protein to an appropriate concentration. SDS-PAGE was used to detect the target proteins after digestion and purification.

### Study on the binding properties of olfactory proteins

HPLC-grade methanol was purchased from Thermo Fisher Scientific, and a fluorescent probe, 1-NPN, was purchased from Sigma. Ten compounds were selected as candidate ligands. Except 3-Octanone, many volatile compounds can have a significant attractive or repellent effect on *M. usitatus* (Tang L. D. et al., 2015a; Li et al., 2021). One study identified 3-Octanone in the seeds of four varieties of legumes, which showed an attractive effect on Callosobruchus maculatus (Adhikary et al., 2015). Standard samples of volatile odorous substances (Supplementary Table S5) were purchased from Shanghai Macleans Biochemical Technology Co., Ltd., China. The fluorescent probe 1-NPN and odor standard sample were dissolved in HPLC-grade methanol solution to a final concentration of 2 μmol/l.

For this experiment, the excitation light wavelength was set to 337 nm, the scanned emission light range was 370–500 nm, and gain was 100. The protein solution was added to a 96-well black microtiter plate at a volume of 250 μl and the fluorescent probe 1-NPN was added to obtain a final concentration of 2 μmol/l. Subsequently, 1-NPN was gradually added until the final concentration of 4–20 μM was reached. The solution was allowed to react for 2 min in the dark, and the maximum fluorescence values were recorded. The Scatchard equation was created using Microsoft Excel to obtain the binding constants (KD values) of MusiOBP1/MusiCSP1 and 1-NPN.

A protein solution (2 μmol/l, 250 μl) was added to a 96-well black microtiter plate, and the fluorescent probe 1-NPN was added to obtain a final concentration of 2 μmol/l. This solution was allowed to react for 2 min in the dark and the maximum fluorescence value was recorded. Next, 2–16 μmol/l odor standards were added individually in the dark. After reacting for 2 min, the maximum fluorescence was recorded. The experiment was repeated three times. The dissociation constants of MusiOBP1, MusiCSP1, and odor molecules were calculated using Eq. 1.

$$Ki=\left({IC}_{50}\right)/\left(1+\left(1-NPN\right)/K\left(1-NPN\right)\right)$$ (1)

where 1-NPN is the concentration of unbound 1-NPN and K (1-NPN) is the binding constant of the MusiOBP1/1-NPN and MusiCSP1/1-NPN complexes.

## Results

### Illumina sequencing and functional annotation results

The samples were sequenced using an Illumina Hiseq 2000. A total of 623,574,528 raw reads were generated from the 4 *M. usitatus* development libraries. After excluding adapters and low-quality sequences from the raw reads, the total number of clean reads from nymphs, pupae, male, and female *M. usitatus* were 134,320,724, 147,280,360, 163,504,704, and 138,063,894, respectively (Supplementary Table S6). A total of 56,669 unigenes obtained using BLAST software were compared using the GO, NR, eggNOG, Swissport, and KEGG databases. According to the NR databases (shown in Supplementary Figure S1), the species distribution chart shows 21,086 annotated unigenes. A total of 7,427 unigenes (35.22%) were annotated as Frankliniella occidentali. Using BLAST2GO software to classify all unigenes according to Gene Ontology, a total of 15,620 unigenes were divided into three categories: biological process, cellular component, and molecular function. A total of 17,987 unigenes were functionally categorized into 26 groups according to eggNOG classification. Except for the “function unknown” category, “signal transduction mechanisms” was the largest functional category with 2,407 unigenes. In total, 10,794 unigenes were annotated to 35 pathways in KEGG level 2, with unigene distribution statistics for each pathway. Among these pathways, unigenes were mainly distributed in signal transduction (14.60%), global and overview maps (13.39%), cancers overview (12.36%), and endocrine system (13.32%) pathways.

### Identification of OBPs and CSPs

**FIGURE 1:**
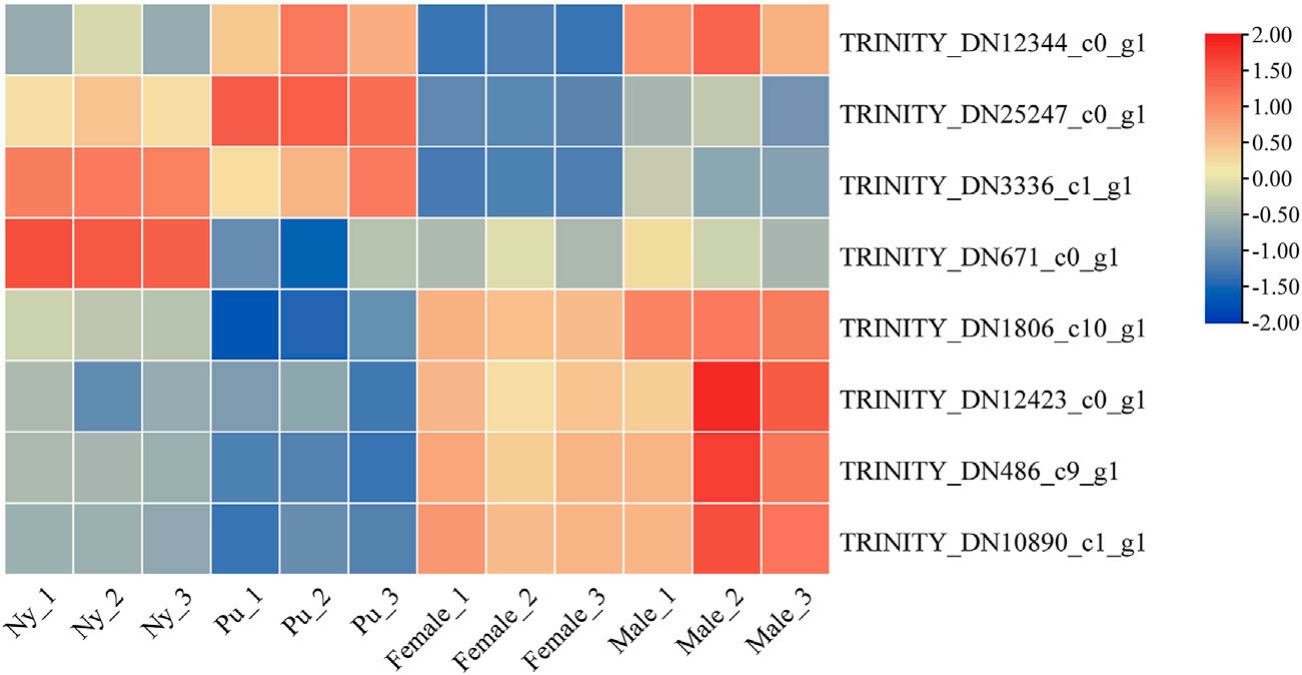
Cluster diagram of OBP and CSP gene expression at different developmental stages.

**FIGURE 2:**
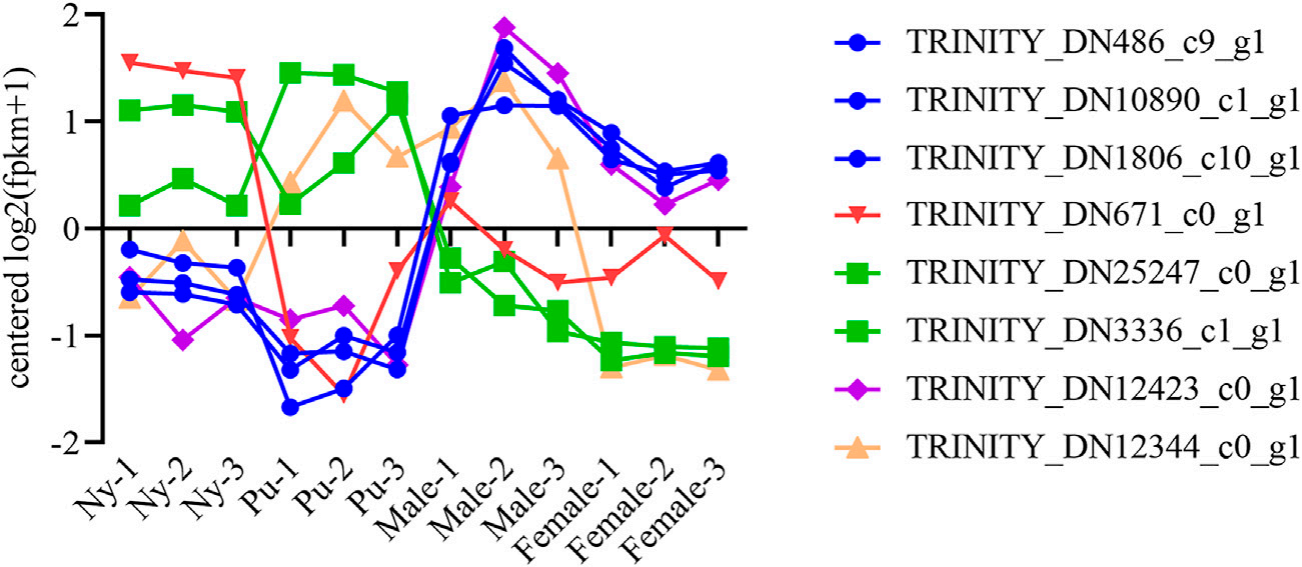
Trend analysis of OBP and CSP genes. Different colors distinguish different clustals.

A total of six OBP and two CSP genes were identified using DEGseq and annotation of odorant-binding proteins and chemosensory proteins (Table 1), and then made into a heat map using TB tools software (Figure 1). The heatmap shows that there were significant differences in OBP and CSP expression levels at different developmental stages. OBP and CSP genes were divided into different clusters according to their expression patterns (the gene expression trend in the same cluster was similar) to obtain gene clustering results. The results are shown in Figure 2.

### Acquisition of full-length sequence of MusiOBP1 and MusiCSP1

Following cloning, colony PCR identification of positive clones was performed. After sequencing, 422 bp at the 3' end and 442 bp at the 5' end of MusiOBP1, 437 bp at the 3' end, and 406 bp at the 5' end of MusiCSP1 were obtained (Supplementary Figure S2).

**FIGURE 3:**
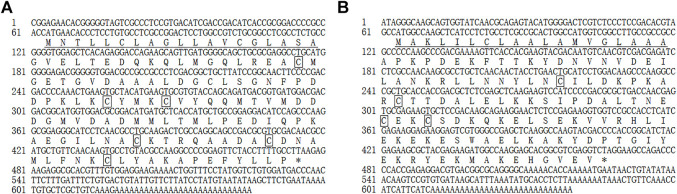
Full-length cDNA sequence of MusiOBP1 **(A)** and MusiCSP1 **(B)**. Underlining indicates the N-terminal signal peptide, the termination codon is marked with *, and the four conserved cysteine sites are marked with boxes.

The full-length MusiOBP1 cDNA sequence length was 645 bp, including a 168 bp 3’ non-coding region and a 63 bp 5’ non-coding region. Sequence analysis showed that the MusiOBP1 open reading frame was 414 bp long, encoding 137 amino acids, with a predicted molecular weight of 14.754 kDa and an isoelectric point of 4.15. MusiOBP1 has six conserved cysteine sites, arranged as ‘C1-X26-C2-X3-C3-X37-C4-X8-C5-X8-C6’, which conforms to the typical model of classical OBPs, “C1-X15-39-C2-X3-C3-X21-44-C4-X7-12-C5-X8-C6,” which in turn conforms to the basic characteristics of insect OBPs (Zhou et al., 2008) (Figure 3A).

The full-length MusiCSP1 cDNA sequence was 639 bp long, including a 171 bp 3’ non-coding region and 63 bp 5’ non-coding region. Sequence analysis revealed that the MusiCSP1 open reading frame was 405 bp, encoding 134 amino acids, with a predicted molecular weight of 15.185 kDa and an isoelectric point of 7.57. MusiCSP1 has four conserved cysteine sites, C1-X8-C2-X18-C3-X2-C4’, which conforms to the typical CSP model, C1-X6-8-C2-X16-21-C3-X2-C4, which in turn conforms to the basic characteristics of insect CSPs (Zhou et al., 2006) (Figure 3B).

### Construction of phylogenetic tree

**FIGURE 4:**
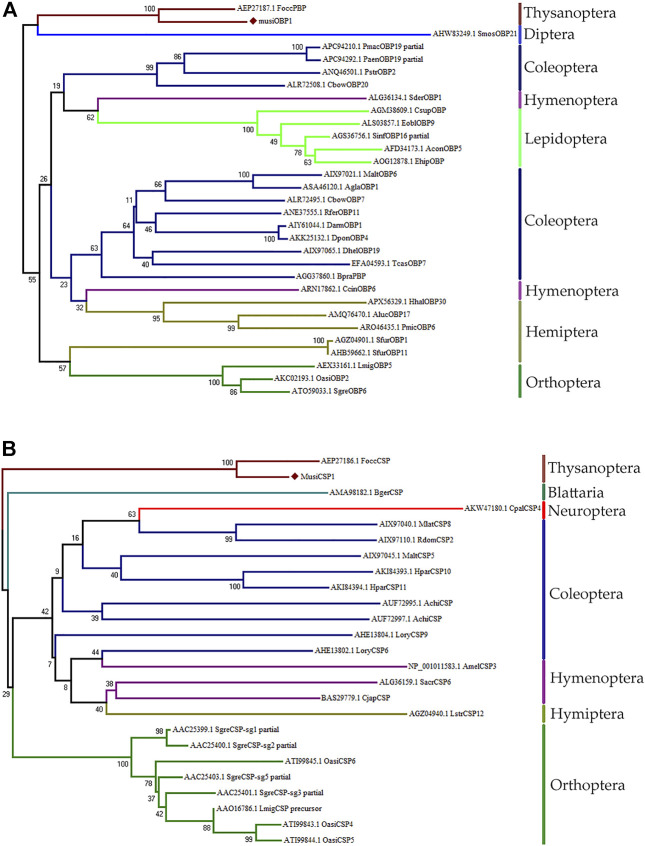
Amino acid sequence phylogenetic tree construction using the NJ method. **(A)** MusiOBP1 and other insect OBPs. **(B)** MusiCSP1 and other insect CSPs.

The NJ tree obtained for MusiOBP1 and MusiCSP1 using MEGA7 is divided into two major branches (Figure 4A). The OBP of *M. usitatus*, Thysanoptera, and Diptera were located in the same branch, indicating that the odor-binding proteins of Thysanoptera and Diptera may have a common ancestral gene. The CSPs of *M. usitatus* and F. occidentalis from Thysanoptera were located in the same branch, indicating that they may have common ancestral genes. *Blattella* has its own branch, indicating that it is genetically distant from other orders (Figure 4B).

### The expression pattern of MusiOBP1 and MusiCSP1

**FIGURE 5:**
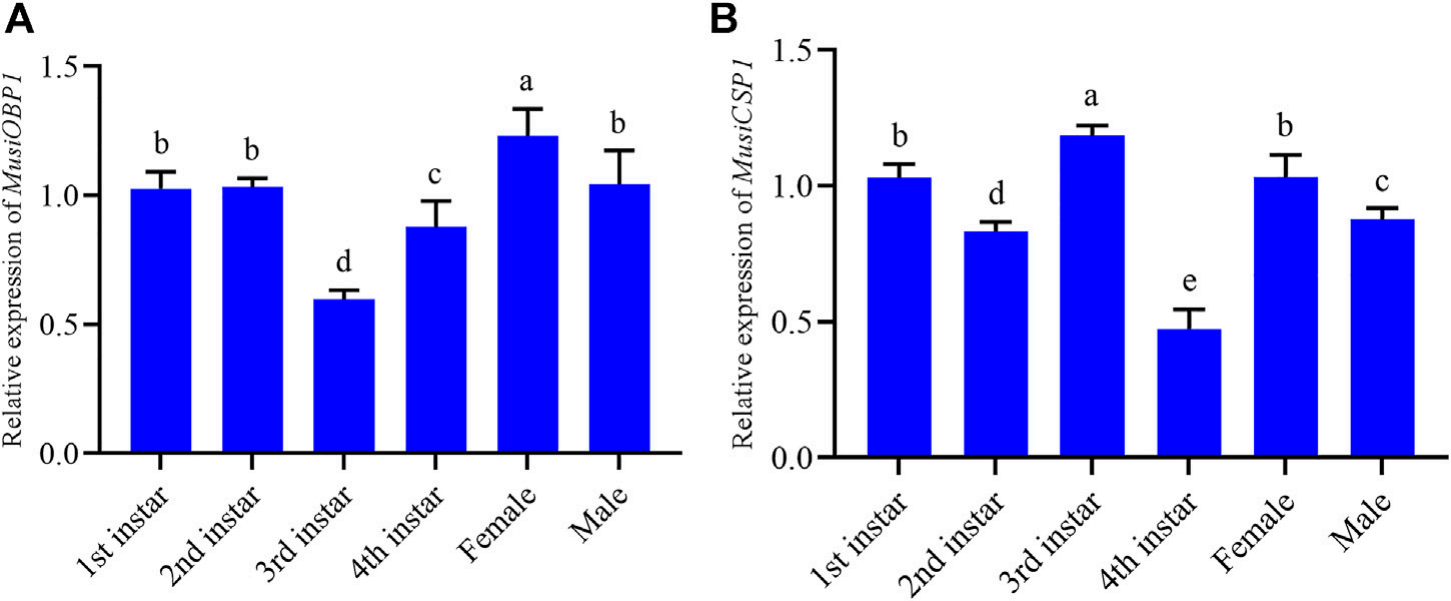
Relative expression levels of MusiOBP1 and MusiCSP1 in different developmental stages of *M. usitatus*. **(A)** MusiOBP1. **(B)** MusiCSP1.

The results show that MusiOBP1 was expressed in all developmental stages of *M. usitatus*, but the degree of expression differed at each stage. The relative expression of MusiOBP1 in newly emerged females was significantly higher than that in other stages (Figure 5A). Furthermore, the relative expression of MusiCSP1 in the pre-pupal stage was significantly higher than that in other developmental stages (Figure 5B). There was no significant difference in the expression of MusiCSP1 between the first instar nymphs and adults.

### Protein expression and purification

MusiOBP1 and MusiCSP1 were successfully expressed in prokaryotic systems. SDS-PAGE analysis revealed that the recombinant proteins pET32a(+)/MusiOBP1 and pET32a(+)/MusiCSP1 had bands of approximately 30 and 31 kDa, respectively, after induction (Supplementary Figure S3A). Ultrasonic fragmentation of induced cultured cells, SDS-PAGE, and western blotting revealed that MusiOBP1 and MusiCSP1 were expressed in the supernatant and inclusion bodies (Supplementary Figure S3B,C). The recombinant protein was purified by affinity chromatography and detected using SDS-PAGE. A large amount of recombinant protein was eluted when 200 mmol/l imidazole elution buffer was used (Supplementary Figure S3D,E).

SDS-PAGE analysis of the purified recombinant proteins showed that the purified target protein had a clean background and a single band (Supplementary Figure S3F). The concentrations of MusiOBP1 and MusiCSP1 were 0.997 and 1.199 mg/ml, respectively.

### The binding ability of proteins to different odor molecules

The binding curve of fluorescent probe 1-NPN with olfactory proteins MusiOBP1 and MusiCSP1 along with the Scatchard equation are shown in Supplementary Figure S4. The fluorescence intensity increased with the addition of 1-NPN. The calculated binding constants of MusiOBP1 and MusiCSP1 were 5.05 μmol/L and 8.36 μmol/L.

**FIGURE 6:**
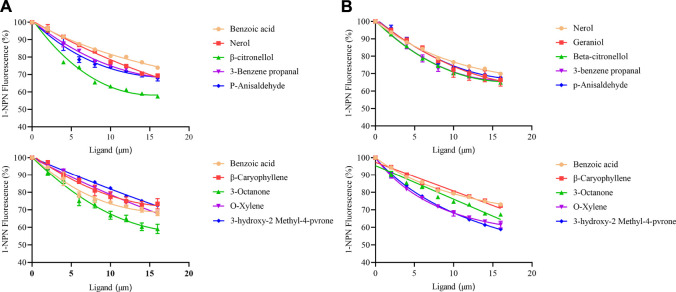
Binding curve of MusiOBP1 and MusiCSP1 with different odor molecules. **(A)** Summary of MusiOBP1 binding affinities for each ligand. **(B)** Summary of MusiCSP1 affinities for each ligand.

A fluorescence competition experiment was used to determine the binding affinity of olfactory proteins MusiOBP1 and MusiCSP1 with ten kinds of odor molecules. The results show that MusiOBP1 had the strongest binding affinity with β-citronellol, with a binding constant of 16.16 μmol/l (Figure 6, Table 2). In contrast, MusiCSP1 showed the strongest binding ability for 3-hydroxy-2-methyl-4-pyrone, with a binding constant of 25.71 μmol/l (Figure 6, Table 2).

**TABLE 2:** Binding characteristics between protein and volatile compounds.

Odor	MusiOBP1		MusiCSP1	
	IC_50_ μmol/l	Ki μmol/l	IC_50_ μmol	Ki μmol/l
Nerol	63.47	48.40	95.79	79.98
Geraniol	73.78	55.94	43.51	36.45
β-citronellol	21.48	16.16	46.25	38.62
P-Anisaldehyde	61.20	46.72	51.52	42.83
3-Benzene propanal	64.57	68.23	46.08	38.60
Benzoic acid	159.25	121.74	157.93	131.65
β-Caryophyllene	77.89	56.72	143.96	120.62
3-Octanone	29.16	20.47	71.06	59.58
O-Xylene	37.27	27.95	37.04	30.99
3-hydroxy-2-Methyl-4-pvrone	110.57	83.53	30.28	25.39

## Discussion

Transcriptome sequencing focuses on functional sites, which represent the most adaptive sites in the genome. It has become a powerful tool for gene discovery, functional identification, gene expression, genetic diversity, and adaptive evolution (Gu et al., 2011; Zhang and Yuan 2013) and it provides an opportunity to explore olfactory genes in insects (Etebari et al., 2011; Wang et al., 2015). Previously, a total of 35,015,872 clean reads were obtained and assembled into 45,800 unigenes by transcriptome sequencing of Cnaphalocrocis medinalis antennae, including 12 OBPs and 15 CSPs (Liu et al., 2017). Following this, developmental transcriptomes of different life stages were built using RNA sequencing in Batocera horsfieldi, and 199 million clean reads were obtained and assembled into 87,732 unigenes. Among these, genes encoding seven OBPs and three CSPs had already been identified (Yang et al., 2018). In the present study, 56,669 unigenes were assembled, and six OBPs and CSPs were identified in the DEGs; the results were similar to those of Batocera horsfieldi developmental transcriptomes. Compared with the antennae transcriptome, developmental transcriptomes produced more clean reads but gained fewer OBPs and CSPs, which may be because OBPs and CSPs are mostly expressed in the antennae and generally have high expression levels (Liu et al., 2020). Transcriptome data were used to study olfactory genes, which helped to elucidate the relationship between olfactory proteins and development, as well as between olfactory proteins and the environment.

Full-length MusiOBP1 and MusiCSP1 sequences were obtained using RACE PCR. MusiOBP1 had six conserved cysteine sites with an isoelectric point of 4.15, whereas MusiCSP1 had four conserved cysteine sites with an isoelectric point of 7.57. MusiOBP1 and MusiCSP1 have common structural signatures with low molecular masses in the OBP and CSP families, respectively (Picimbon et al., 2000; Zhou et al., 2008). The NJ tree showed that MusiOBP1 and MusiCSP1 were present in the groups with only FoccPBP and FoccCSP, respectively. This suggests that MusiOBP1 and MusiCSP1 are highly homologous to FoccPBP and FoccCSP, respectively.

This variable expression suggests that this gene may have different roles at different stages of development. The RT-qPCR results show that MusiOBP1 and MusiCSP1 were expressed at various levels at all developmental stages, indicating that MusiOBP1 and MusiCSP1 have a wide range of developmental expression profiles. This is in agreement with previous studies, which demonstrated that OBP and CSP can be differentially expressed at different stages, such as in larvae, pupae, and adults (He et al., 2011; Zhang et al., 2020b). MusiOBP1 generally has the highest expression levels in early emergence females; OBPs are significantly upregulated in the adult phase of many insects, such as the genes PxylOBP6 and PxylOBP11 in Plutella xylostella (Yang et al., 2017), and BdOBP56d, BdOBP99a, BdOBP99c, and BdOBP19 in Bactrocera dorsalis (Zhang et al., 2018). In common large thrips this may be related to their habit of entering the soil during the pre-and pseudo-pupal stages, while adults must search for host plants to obtain nutrients and carry out oviposition (Han et al., 2015). Furthermore, once females were exposed to their chosen host (banana or orange), all OBP expression levels increased, but when exposed to bitter gourd or tomato, some OBP expression levels (OBP19c and OBP44a) decreased (Zhang et al., 2018), indicating that OBP expression is linked to the host; high expression levels of MusiOBP1 in females may also be a contributing factor. MusiCSP1 had the highest relative expression level in the pre-pupal stage, which is the transitional stage from the active nymph to the pupal stage. A previous study showed that CresCSP8 is highly expressed in the forth and fifth nymphs of Clostera restitura (Li H. et al., 2019b), and that CSP1 is the most highly expressed gene in the egg and pupal stages of Anomala corpulenta (Chen et al., 2019). It has been hypothesized that chemosensory protein genes are related to growth and development, which would align with the expression pattern of MusiCSP1.

Many volatile compounds (except for 3-Octanone) can have a significant attractive or repellent effect on *M. usitatus* (Tang L. D. et al., 2015a; Li et al., 2021). One study identified 3-Octanone in the seeds of four varieties of legumes, which were attractive to Callosobruchus maculatus (Adhikary et al., 2015). The most widely used fluorescent probe for competitive fluorescence binding in insect OBPs and CSPs is 1-NPN, such as Anoplophora glabripennis AglaOBP1 (Li et al., 2017) and Grapholita molesta GmolOBP14 (Chen X. L. et al., 2018a). In this study, MusiOBP1 and MusiCSP1 had good binding affinities with 1-NPN. Similar to research on DsOBP69a and DsOBP76a (Zhan et al., 2021), after adding 1-NPN and the protein solution, we added 2–16 μmol/L odor molecules to determine the dissociation constants. Although at 16 μmol/L, the compound with the strongest binding affinity to proteins can only reduce the fluorescence intensity to < 60%, IC50 and Ki calculations can reflect the binding affinity of proteins with each odor molecule. Fluorescence binding assays revealed that the target protein, MusiOBP1, binds strongly to β-citronellol. The target proteins MusiCSP1 and 3-Hydroxy-2-methyl-4-pyrone also have strong binding affinities. *Apis ceranacerana* odorant-binding protein AcerOBP15 has a strong binding affinity for β-citronellol, suggesting that AcerOBP15 plays a role in orientation to food sources (Du et al., 2021). β-citronellol can be extracted from a range of plants, including *M. usitatus*’ host, tobacco (Yang et al., 2015; Chen L. et al., 2018b). MusiOBP1 binds well to alcohols, aldehydes, ketones, and alkenes but not to acids. This may be due to differences in the ligand structure, and further research is needed to confirm this. 3-Hydroxy-2-methyl-4-pyrone can be extracted from another *M. usitatus* host, cowpea flowers (Li et al., 2021), indicating that MusiOBP1 and MusiCSP1 may participate in host identification and localization. It is likely that DEET and other insect repellents are delivered to their particular receptors via OBPs, as has been demonstrated for other odorants (Biessmann et al., 2010). β-citronellol demonstrated comparable interactions with the OBP4 binding site as DEET (Mota et al., 2022), suggesting that β-citronellol can act as a potential *M. usitatus* repellent.

Fluorescence binding assays suggested that MusiOBP1 and MusiCSP1 are involved in the recognition, binding, and transport of various compounds. However, the exact physiological roles of MusiOBP1 and MusiCSP1 remain to be elucidated. Much work remains to better understand the functions of *M. usitatus* OBP and CSP families. This is the first study on the olfactory protein gene of *M. usitatus*, which provides a reference for future studies on the function of olfactory protein genes as well as new direction for the prevention and control of *M. usitatus* through the olfactory system.

## Conclusion

In summary, RT-qPCR revealed high levels of MusiOBP1 expression in females and high MuisCSP1 expression levels in pupae. Fluorescence binding assays showed that MusiOBP1 has a strong binding affinity for β-citronellol and MusiCSP1 binds strongly to 3-hydroxy-2-methyl-4-pvrone. Our study indicates that MusiOBP1 and MusiCSP1 may play an important role in mediating *M. usitatus* host recognition, which provides a reference for a follow-up study on the function of olfactory protein genes and a new direction for the prevention and control of *M. usitatus* through the olfactory system.

## Data Availability

The original contributions presented in the study are publicly available. This data can be found here: https://www.ncbi.nlm.nih.gov/sra/?term=PRJNA877671.
