# Supplementary figures and images for: Transcriptome analysis of megalurothrips usitatus (Bagnall) identifies olfactory genes with ligands binding characteristics of MusiOBP1 and MusiCSP1
Source: Front Physiol. 2022 Sep 26;13:978534. doi: 10.3389/fphys.2022.978534 (PMC9549282; doi:10.3389/fphys.2022.978534)

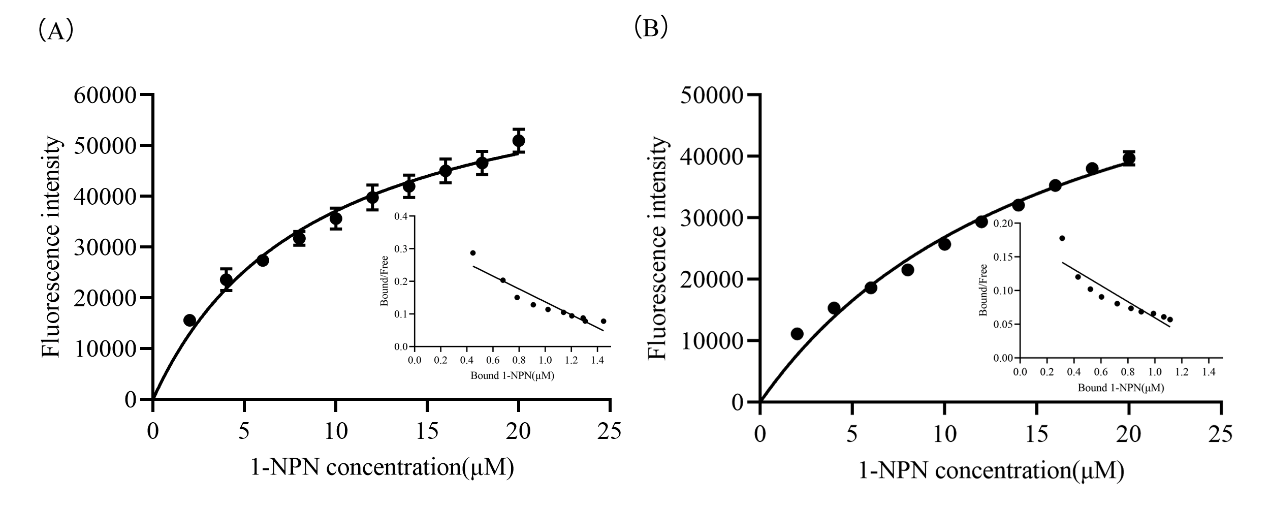


**Supplementary Figure S4**Binding curve of 1-NPN and Scatchard equation. (A) MusiOBP1, (B) MusiCSP1*.*

Supplement: Supplementary file 1 [file DataSheet4.docx]
