# Supplementary material for: Transcriptome analysis of megalurothrips usitatus (Bagnall) identifies olfactory genes with ligands binding characteristics of MusiOBP1 and MusiCSP1
Source: Front Physiol. 2022 Sep 26;13:978534. doi: 10.3389/fphys.2022.978534 (PMC9549282; doi:10.3389/fphys.2022.978534)
Supplement: Supplementary file 2 [file Table1.DOCX]

Supplementary Table 1 Primers for amplification of known sequences of MusiOBP1 and MusiCSP1

| Primer name | Sequence（5’-3’） | annealing temperature |
| --- | --- | --- |
| MusiOBP1-F | GACTCCTGGCCGTCTGC | 60℃ |
| MusiOBP1-R | TTGACAGCGAGCACATTTTA |  |
| MusiCSP1-F | CCCGACGAAAAGTTCACCAC | 60℃ |
| MusiCSP1-R | TGCTTATCACACGGACTTGTTT |  |
