# Supplementary material for: Transcriptome analysis of megalurothrips usitatus (Bagnall) identifies olfactory genes with ligands binding characteristics of MusiOBP1 and MusiCSP1
Source: Front Physiol. 2022 Sep 26;13:978534. doi: 10.3389/fphys.2022.978534 (PMC9549282; doi:10.3389/fphys.2022.978534)
Supplement: Supplementary file 3 [file DataSheet3.docx]

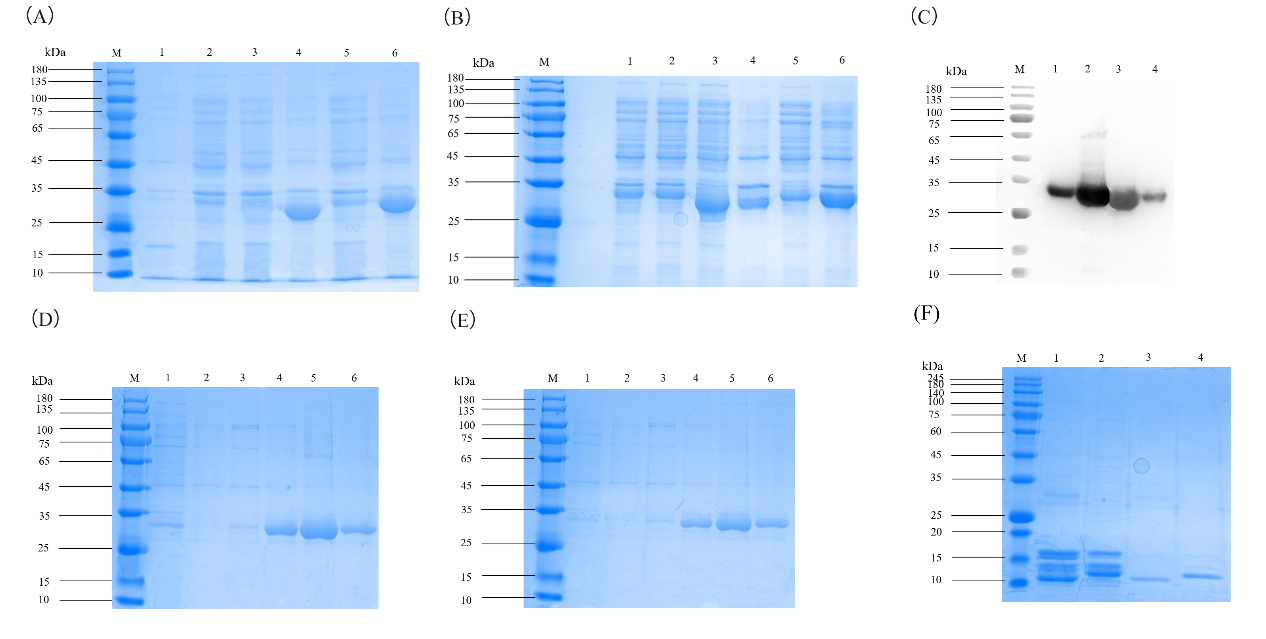


**Supplementary Figure S3** Protein expression and purification

Note: (A): M: Protein Marker; 1: Uninduced empty plasmid, 2: iduced empty plasmid; 3: The recombinant expression vector pET32a(+)/MusiOBP1 before induction; 4: The induced recombinant expression plasmid pet32a(+)/MusiOBP1; 5: The recombinant expression vector pET32a(+)/MusiCSP1 before induction; 6: The induced recombinant expression plasmid pET32a(+)MusiCSP1.

(B): M: Protein Marker; 1: recombinant expression plasmid pet32a(+)/MusiOBP1 before induction 2: recombinant expression plasmid pet32a(+)/MusiCSP1 before inductionAfter induction; 3: the recombinant expression plasmid pET32a(+)/MusiOBP1 was supernatant; 4: After induction, the recombinant expression plasmid pET32a(+)/MusiOBP1 was precipitated; 5: After induction, the recombinant expression plasmid pET32a(+)/MusiCSP1 was supernatant; 6: After induction, the recombinant expression plasmid pET32a(+)/MusiCSP1was precipitated.

(C): M: Protein Marker; 1: the recombinant expression plasmid pET32a(+)/MusiCSP1 was supernatant; 2: After induction, the recombinant expression plasmid pET32a(+)/MusiCSP1 was precipitated; 3: After induction, the recombinant expression plasmid pET32a(+)/MusiOBP1 was supernatant; 4: After induction, the recombinant expression plasmid pET32a(+)/MusiOBP1 was precipitated.

(D): M: Protein Marker; 1: Flow through liquid after protein binding with Ni column; 2: The Ni column was washed with 10 mmol / L imidazole eluent; 3, 4, 5, 6: MusiOBP1 eluted with 50, 100, 200, 300 mmol / L imidazole eluent.

(E): M: Protein Marker; 1: Flow through liquid after protein binding with Ni column; 2: The Ni column was washed with 10 mmol / L imidazole eluent; 3, 4, 5, 6: MusiCSP1 eluted with 50, 100, 200, 300 mmol / L imidazole eluent.

(F): M: protein Marker; 1: The recombinant protein pet32a(+)/MusiOBP1 after enzyme digestion; 2: The recombinant protein pet32a (+) / MusiCSP1 after enzyme digestion; 3: Purified MusiOBP1 protein; 4: Purified MusiCSP1 protein.
