# Supplementary material for: Transcriptome analysis of megalurothrips usitatus (Bagnall) identifies olfactory genes with ligands binding characteristics of MusiOBP1 and MusiCSP1
Source: Front Physiol. 2022 Sep 26;13:978534. doi: 10.3389/fphys.2022.978534 (PMC9549282; doi:10.3389/fphys.2022.978534)
Supplement: Supplementary file 4 [file Table2.DOCX]

Supplementary Table 2 Primer information for RACE PCR

| Gene name | Primer  name | Sequence  （5’-3’） | Annealing  temperature |
| --- | --- | --- | --- |
| MusiOBP1 | 3’MusiOBP1-Outer | ACAGAGGACCAGAAGCAGTTGATGGGGCAG | 68℃ |
|  | 3’MusiOBP1-Inner | AGATGACGGTGATGGACGACGACGGCATG | 68℃ |
|  | 5’MusiOBP1-Outer | AGTTGGGTCATCCACAGACCATAGG | 65℃ |
|  | 5’MusiOBP1-Inner | ACAGCATGGCGTTGTCGCACG | 65℃ |
| MusiCSP1 | 3’MusiCSP1-Outer | CCCCCAAGCCCGACGAAAAGT | 62℃ |
|  | 3’MusiCSP1-Inner | CAAGCCCAAGGCCCGCTG | 65℃ |
|  | 5’MusiCSP1-Outer | CGTCACGTCCTCTCGGTGGG | 64℃ |
|  | 5’MusiCSP1-Inner | GCTCGGCCCACGACTCCTTCT | 65℃ |
| UPM | | CTAATACGACTCACTATAGGGCAAGCAGTGGTATCAACGCAGAGT | ∕ |
| NUP | | CTAATACGAC TCACTATAGGGC | ∕ |
