# Supplementary material for: Transcriptome analysis of megalurothrips usitatus (Bagnall) identifies olfactory genes with ligands binding characteristics of MusiOBP1 and MusiCSP1
Source: Front Physiol. 2022 Sep 26;13:978534. doi: 10.3389/fphys.2022.978534 (PMC9549282; doi:10.3389/fphys.2022.978534)
Supplement: Supplementary file 5 [file Table5.docx]

Supplementary Table 5 Standard samples of odor volatile substances

| **Odor** | **Purity（%）** | **CAS number** |
| --- | --- | --- |
| Nerol | 97 | 106-25-2 |
| Geraniol | 98 | 106-24-1 |
| Beta-Citronellol | 95 | 106-22-9 |
| P-Anisaldehyde | 99 | 123-11-5 |
| 3-benzene propanal | 95 | 104-53-0 |
| Benzoic acid | 99 | 65-85-0 |
| Beta-caryophyllene | 80 | [87-44-5](http://www.macklin.cn/search/87-44-5) |
| 3-Octanone | 98 | [106-68-3](http://www.macklin.cn/search/106-68-3) |
| O-Xylene | 98 | 95-47-6 |
| 3-hydroxy-2 Methyl-4-pvrone pvrone | 99 | 118-71-8 |
