# Supplementary material for: Transcriptome analysis of megalurothrips usitatus (Bagnall) identifies olfactory genes with ligands binding characteristics of MusiOBP1 and MusiCSP1
Source: Front Physiol. 2022 Sep 26;13:978534. doi: 10.3389/fphys.2022.978534 (PMC9549282; doi:10.3389/fphys.2022.978534)
Supplement: Supplementary file 6 [file Table3.docx]

Supplementary Table 3 Primers for RT-qPCR

| Gene name | Sequence（5’-3’） |
| --- | --- |
| qMusiOBP1-F | TTCAACAAGTGCCTGTACGC |
| qMusiOBP1-R | GGTCATCCACAGACCATAGGA |
| qMusiCSP1-F | CTACCTGAACTGCATCCTGGACAAG |
| qMusiCSP1-R | GAGCACTTCTCGCACTCGTTGG |
| qGAPDH-F | ACTGTTGATGGTCCCTCTGG |
| qGAPDH-R | AGCGGCTTCCTTAACCTTCT |
