# Supplementary material for: Transcriptome analysis of megalurothrips usitatus (Bagnall) identifies olfactory genes with ligands binding characteristics of MusiOBP1 and MusiCSP1
Source: Front Physiol. 2022 Sep 26;13:978534. doi: 10.3389/fphys.2022.978534 (PMC9549282; doi:10.3389/fphys.2022.978534)
Supplement: Supplementary file 7 [file DataSheet2.docx]

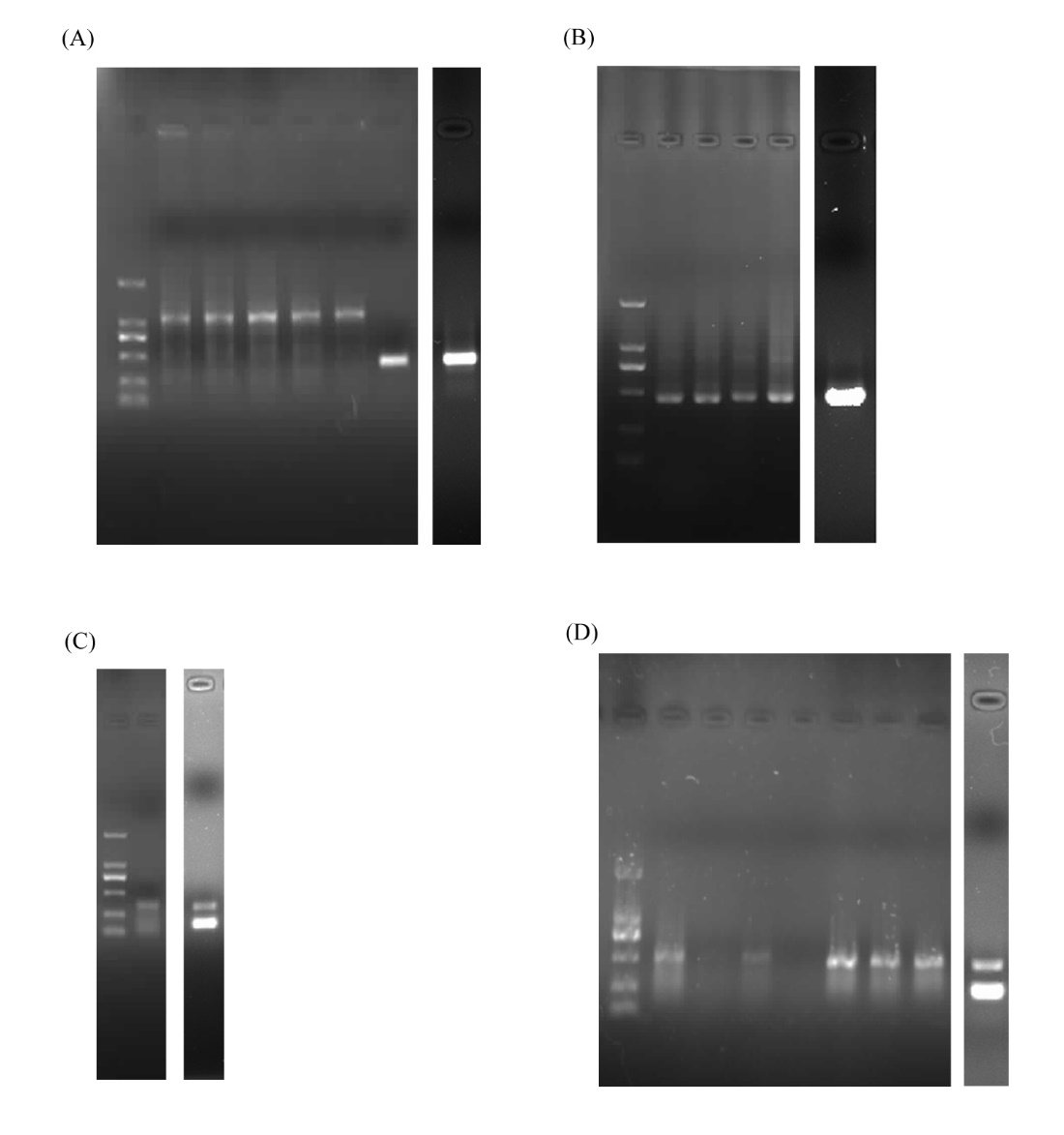


**Supplementary Figure S2** RACE PCR amplification of *M. usitatus* olfactory protein genes. A: Amplification results of MusiOBP1 3' RACE PCR. B: Amplification results of MusiCSP1 3' RACE PCR. C: Amplification results of MusiOBP1 5' RACE PCR. D: Amplification results of MusiCSP1 5' RACE PCR.
