# Supplementary material for: Transcriptome analysis of megalurothrips usitatus (Bagnall) identifies olfactory genes with ligands binding characteristics of MusiOBP1 and MusiCSP1
Source: Front Physiol. 2022 Sep 26;13:978534. doi: 10.3389/fphys.2022.978534 (PMC9549282; doi:10.3389/fphys.2022.978534)
Supplement: Supplementary file 8 [file Table4.docx]

Supplementary Table 4 Primers for amplification of the ORF of genes

| Gene name | Sequence（5’-3’） |
| --- | --- |
| MusiOBP1-F | ataGGATCCGGTGGAGCTCACAGAGGACC |
| MusiOBP1-R | gcAAGCTTTTAAGGCAAAAGGTAGAACT |
| MusiCSP1-F | ataGGATCCGCCCCCAAGCCCGACGAAAA |
| MusiCSP1-R | attAAGCTTCTAGACCTCGACGCCGTGCT |
