# Supplementary material for: Transcriptome analysis of megalurothrips usitatus (Bagnall) identifies olfactory genes with ligands binding characteristics of MusiOBP1 and MusiCSP1
Source: Front Physiol. 2022 Sep 26;13:978534. doi: 10.3389/fphys.2022.978534 (PMC9549282; doi:10.3389/fphys.2022.978534)
Supplement: Supplementary file 9 [file Table6.docx]

Supplementary Table 6 Summary of sequencing data of four cDNA samples of *M. usitatus*

| Type | Nymphal | Pupa | Male | Female | Total |
| --- | --- | --- | --- | --- | --- |
| Total number of raw reads | ‬143583338 | 157690512 | 174378186‬ | 147922492 | 633574528 |
| Total number of clean reads | 134320724 | 147280360 | 163504704 | 138063894 | 583169682 |
| Clean Reads (%) | 93.55 | 93.40 | 93.76 | 93.34 | 93.52 |
