# Supplementary material for: Transcriptome analysis of megalurothrips usitatus (Bagnall) identifies olfactory genes with ligands binding characteristics of MusiOBP1 and MusiCSP1
Source: Front Physiol. 2022 Sep 26;13:978534. doi: 10.3389/fphys.2022.978534 (PMC9549282; doi:10.3389/fphys.2022.978534)
Supplement: Supplementary file 10 [file DataSheet1.docx]

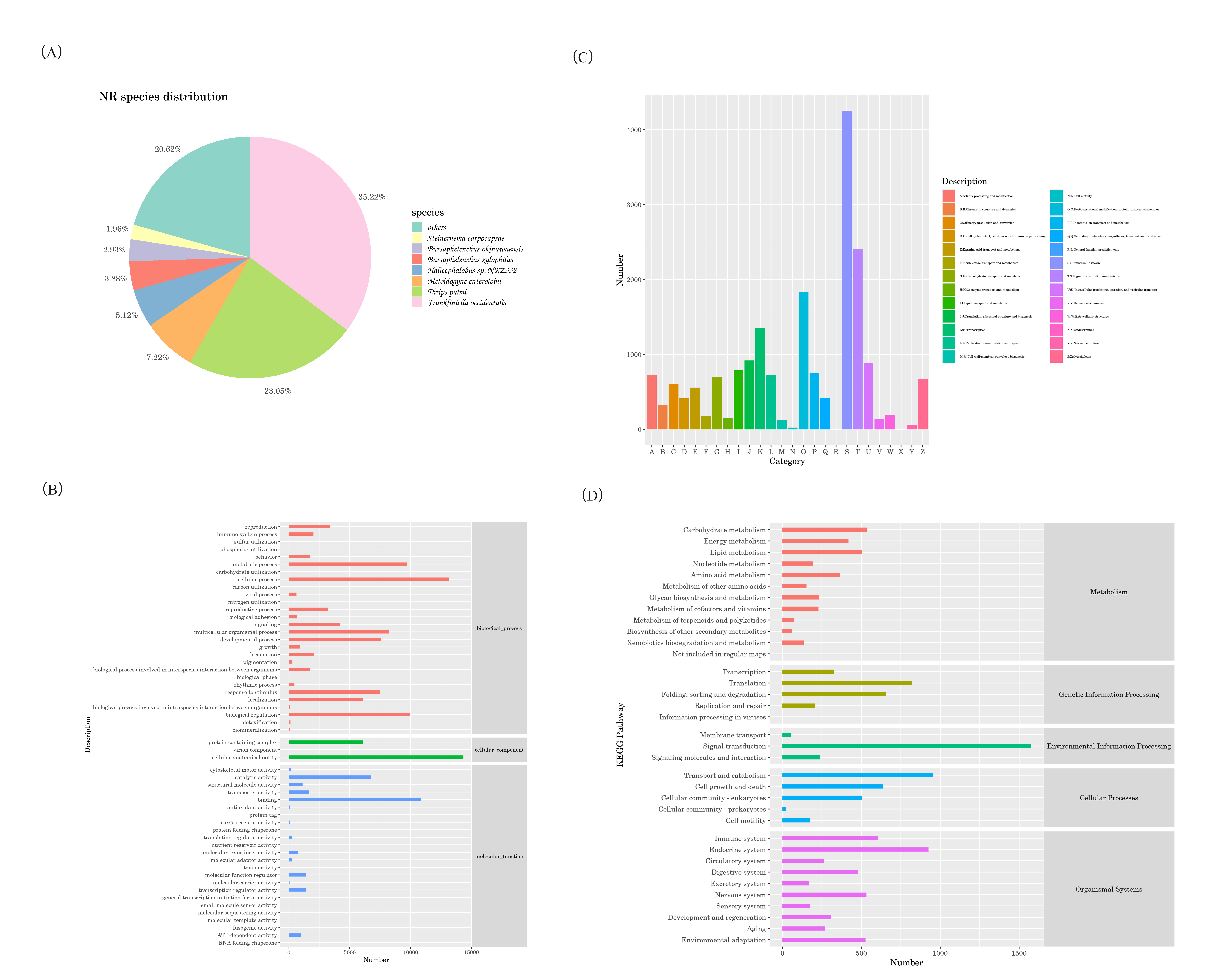


**Supplementary Figure S1** Gene function annotation of Unigene. (A) NR annotation statistics. (B) GO annotation statistics .(C) eggNOG amnotation statistics. (D) KEGG amnotation statistics
